# Guided parent-delivered cognitive behavioral therapy for childhood anxiety: Predictors of treatment response

**DOI:** 10.1016/j.janxdis.2016.11.003

**Published:** 2017-01

**Authors:** Kerstin Thirlwall, Peter Cooper, Cathy Creswell

**Affiliations:** a School of Psychology and Clinical Language Sciences, University of Reading, UK; b Department of Psychology, Stellenbosch University, South Africa

**Keywords:** Anxiety disorders, Cognitive behavior therapy, Child/adolescent, Treatment, Stepped-care, Prediction of response

## Abstract

**Background:**

Guided Parent-delivered Cognitive Behaviour Therapy (GPD-CBT) is a brief, effective treatment for childhood anxiety disorders, however not all children respond favourably.

**Aims:**

To examine predictors of response to GPD-CBT.

**Methods:**

Parents of 125 children (7–12 years) with an anxiety disorder received GPD-CBT over 2.6 or 5.3 h. Recovery was measured post treatment and six months later.

**Results:**

Younger children and those with primary Generalised Anxiety Disorder (GAD) improved more post treatment, but older children and those without primary GAD had better outcomes at six month follow up. Fewer children allocated to 2.6 h had recovered post treatment compared to those allocated to the 5.2 h intervention, but did not differ significantly six months later.

**Conclusions:**

The identification of predictors of short and longer-term treatment outcomes can guide treatment decisions following this low-intensity approach.

## Highlights

• We examined predictors of response to low intensity treatment of childhood anxiety disorders.
• Response was measured at two time points; post treatment and six month follow up.
• Recovery was associated with child age, primary diagnosis of Generalized Anxiety Disorder (GAD) and treatment intensity.
• The findings inform decision making about when to consider more intensive treatment.

## 1 Introduction

Childhood anxiety disorders are common and negatively impact healthy development (Ezpeleta, Keeler, & Erkanli, 2001; Ford, Goodman, & Meltzer, 2003; Polanczyk, Salum, & Sugaya, 2015). Notably, they are associated with persistent difficulties and present a risk for further psychological disturbance and adversity in later life (Bittner, Egger, & Erkanli, 2007). There is consistent support for the use of Cognitive Behavior Therapy (CBT) in treating anxiety disorders in children (James, James, & Cowdrey, 2013; Reynolds, Wilson, & Austin, 2012), however a large proportion of children (approx. 40%) do not recover following this treatment approach (Reynolds et al., 2012). Furthermore traditional delivery of CBT is considered to be relatively resource intensive (Walkup, Albano, & Piacentini, 2008) which, in combination with the high prevalence of anxiety disorders (Kessler, Chiu, & Demler, 2005), means that many children who might benefit are left untreated (Farmer, Stangl, & Burns, 1999; Stallard, Udwin, & Goddard, 2007).

Guided Parent-Delivered CBT (GPD-CBT) is a low intensity form of CBT that requires less therapist contact and fewer resources than standard forms of CBT for childhood anxiety disorders (Lyneham & Rapee, 2006; Rapee, Abbott, & Lyneham, 2006; Smith, Flannery-Schroeder, & Gorman, 2014; Thirlwall, Cooper, & Karalus, 2013). This approach involves parents being guided in implementing CBT strategies in their child’s day to day life and has been shown to be an effective treatment for anxiety disorders in children (Chavira, Drahota, & Garland, 2014; Lyneham and Rapee, 2006, Smith et al., 2014, Thirlwall et al., 2013) with similar outcomes to those found from more intensive CBT delivered face to face with children and parents (Chavira et al., 2014; Cobham, 2012; Leong, Cobham, & De Groot, 2009). As such, GPD-CBT lends itself well to a possible ‘stepped care’ service model, in which low-intensity treatments, which use substantially fewer resources than conventional treatments (Salloum, 2010), are routinely administered and more intensive treatments are reserved for those who may require more specialist input (Bower & Gilbody, 2005). The success of a stepped care model of service delivery is, however, reliant upon clinicians making informed decisions regarding suitability for low-intensity treatment and ‘stepping up’ service users to higher intensity treatments when warranted.

There is currently no information available to guide clinicians and service providers in making decisions about when brief GPD-CBT may or may not be an appropriate treatment.

Few clinical or demographic features reliably predict outcomes from standard child-focused CBT for children with anxiety disorders (Knight, McLellan, Jones, & Hudson, 2014; Lundkvist-Houndoumadi, Hougaard, & Thastum, 2014) and no studies to date have examined predictors of outcome from GPD-CBT specifically. Among studies of standard child-focused CBT for child anxiety disorders, the most consistent predictor of treatment outcome is higher baseline symptom severity (Compton, Peris, & Almirall, 2014; Last, Hansen, & Franco, 1998; Liber, van Widenfelt, & van der Leeden, 2010). Three is also some evidence that co-morbid mood and externalizing disorders are associated with poorer treatment outcome (Berman, Weems, Silverman, & Kurtines, 2000; Hudson, Keers, & Roberts, 2015; Rapee et al., 2013). Recent relatively large treatment studies have also identified other potentially important diagnostic factors. For example, the findings from two large multi-site studies (The ‘Child/Adolescent Anxiety Multimodal Study’ and the ‘Genes for Treatment Study’) indicated that a principal diagnosis of social anxiety disorder was associated with less favorable treatment outcomes from CBT delivered across a range of formats (Compton et al., 2014, Hudson et al., 2015). Consistent with some previous findings (Rapee et al., 2013), the latter study also identified co-morbid mood and externalizing disorders as predictors of poorer treatment outcome. Whether these factors specifically predict treatment outcomes following low-intensity parent-delivered CBT for childhood anxiety disorders remains unclear.

The current study is an examination of predictors of treatment response in a randomized trial of GPD-CBT for the treatment of childhood anxiety disorders in the absence of current maternal anxiety (Thirlwall et al., 2013). The trial sought to examine two versions of GPD-CBT with varying levels of therapist contact to a wait-list control group in order to clarify the level of guidance required for this approach to be effective. Thus the trial involved the delivery of ‘full GPD- CBT’, with approximately 5 h 20 min of therapist contact or ‘brief GPD-CBT’ with approximately 2 h 40 min of therapist contact. Both forms of the treatment were delivered over eight weeks. Participants were 194 children with a primary anxiety disorder diagnosis (159 completers). Intention-to-treat analyses showed that full GPD-CBT produced statistically significant superior diagnostic outcomes (50% free of primary diagnosis) to the wait-list (25%) post treatment, but brief GPD-CBT did not (39%). All results from sensitivity analyses (per protocol, adjusting for minimisation criteria and using multiple imputation) were very similar to the main results. In the current study potential predictors were evaluated on the basis that previous studies have suggested a (albeit not consistent) significant association with outcome from CBT for child anxiety disorders and that the information is typically easily available to inform clinical decision making. As such, we investigated associations between treatment outcome and child demographic characteristics, anxiety severity, the presence of particular anxiety disorder diagnoses as the primary diagnosis or anywhere in the diagnostic profile, co-morbidity of anxiety disorders, the presence of low mood and co-morbidity with behavioral disorders. The two levels of treatment intensity were also examined as predictors of treatment response. Given the inconsistencies in outcome measures used in previous research and in an attempt to provide data that can be compared against other studies, treatment success was determined on the basis of two measures that have most commonly been used as the primary outcome in recent treatment trials for anxiety disorders in children: recovery from primary diagnosis and recovery from all anxiety diagnoses (Cobham, Dadds, & Spence, 2010; Hudson, Rapee, & Deveney, 2009; Kendall, Hudson, & Gosch, 2008; Salloum, 2010). A final methodological consideration was the inclusion of outcome assessments conducted both post-treatment and at a six-month follow-up given that the association between certain predictors and outcome has been found to differ according to when outcome is measured (Hudson et al., 2015). This consideration is particularly pertinent in relation to low intensity treatments where decisions will need to be made about whether and when children should be ‘stepped up’ to a more intensive treatment approach (e.g. individual or group child- focused CBT). As many children recover from brief treatment in the six months after treatment ends (Thirlwall et al., 2013) it is important to know which children are, and are not, likely to make further gains beyond the end of a low intensity treatment in order to know whether to initially monitor progress or to offer an alternative treatment straight away.

## 2 Methods

### 2.1 Sample

The study comprises data from 125 clinically anxious children, aged 7–12 years who were referred from local health and education services and allocated to receive either a 5 h 20 min GPD-CBT treatment (n = 64) or a 2 h 40 min GPD-CBT treatment (n = 61) as part of a randomized control trail examining the efficacy of two versions of GPD-CBT with varying levels of therapist contact to a wait-list control group (Thirlwall et al., 2013) Inclusion criteria for the RCT dictated that children did not have a significant physical or intellectual impairment (including autism spectrum disorders) and that the primary carer did not have a current DSM-IV anxiety disorder or other severe mental health difficulty. The sample represented children with a broad range of anxiety disorders with a range of severity. The majority of primary carers were married, had completed further education and had ‘higher professional’ socioeconomic status. Graduate psychologists systematically assessed all children and their primary carer to establish suitability for the RCT and to obtain baseline measures (see below).

### 2.2 GPD-CBT treatment

Parents were given a self-help book (Creswell & Willetts, 2007) and allocated to receive either weekly therapist contact over eight weeks, involving four 1-h face-to-face sessions and four 20-min telephone sessions (i.e. 5 h and 20 min of therapist guidance) or fortnightly therapist contact over eight weeks, involving two 1-h face-to-face sessions and two 20-min telephone sessions (i.e. 2 h and 40 min of therapist guidance). The role of the therapist was to support and encourage parents to work through the self-help book, rehearse skills with their child at home and to discuss any difficulties that arose.

As is common for low-intensity treatments, the therapists who delivered the treatment had varying levels of clinical experience and were categorized as either having ‘some CBT clinical experience’ (n = 10) or as being ‘novices’ (n = 9). Of importance to this study, there were no significant differences in child treatment outcomes on the basis of therapist experience (Thirlwall et al., 2013). All therapists received weekly, 2-h group supervision with a clinical psychologist (KT) and all treatment sessions were audio recorded and monitored at regular intervals to check for adherence to treatment delivery. Rigorous checks were made on treatment content and treatment fidelity was confirmed (Thirlwall et al., 2013).

### 2.3 Measures

#### 2.3.1 Anxiety disorders interview schedule

The presence and severity of childhood anxiety disorders, as well as mood and behavioral disorders, was assessed using the Anxiety Disorders Interview Schedule for DSM-IV: Child and Parent version (ADIS-C/P) (Silverman & Albano, 1996), a structured diagnostic interview with well-established psychometric properties (Silverman, Saavedra, & Pina, 2001). As is standard, where children met symptom criteria for a diagnosis, the diagnosis with the highest clinical severity rating (CSR) was classed as the primary diagnosis. Each assessor discussed at least their first 20 interviews with a consensus team led by an experienced diagnostician (Consultant Clinical Psychologist) and were required to attain reliability at a kappa/intraclass correlation of 0.85. Once this level of reliability had been reached, assessors discussed one in six interviews with the consensus team, in order to prevent rater drift. Overall inter-rater reliability for the assessor team was excellent (child-report diagnosis: kappa = 0.98; CSR: ICC = 0.98; parent-report diagnosis: kappa = 0.98; CSR: ICC = 0.97).

#### 2.3.2 Spence children’s anxiety scale

Parent and child reports of symptom severity were measured using the Spence Children’s Anxiety Scale: Child and Parent versions (SCAS-C/P) (Spence, 1998). The SCAS consists of 38 items that are rated on 4-point scales to indicate the degree to which the symptoms of anxiety apply to the child (never, sometimes, often and always). The measure has been validated for use with children aged from six years and found to have good reliability, as well as discriminant and convergent validity (Nauta, Scholing, & Rapee, 2004). In the current study, good internal consistency was obtained (SCAS-C: α = 0.87 SCAS-P: α = 0.90).

#### 2.3.3 Short mood and feelings questionnaire

Symptoms of low mood were assessed via child self-report using the Short Mood and Feelings Questionnaire (SMFQ-C) (Angold, Costello, & Messer, 1995). The SMFQ-C consists of 11 core depressive symptom items that are rated on 3-point scales to indicate whether or not the symptoms apply to the child (true, sometimes true, not true). The SMFQ-C has demonstrated high concurrent validity (Angold et al., 1995) and good internal consistency and predictive validity with children from seven years of age (Sharp, Goodyer, & Croudace, 2006). Internal consistency in the current study was good (α = 0.80) (Table 1).

### 2.4 Analytic strategy

In order to restrict the number of variables in multivariate analyses, bivariate associations between each potential predictor and outcome measure were first explored using point-biserial correlations for continuous predictors and Chi-square tests for associations between outcome and dichotomous predictors (see Table 2). We ran all analyses using two treatment outcomes: recovery from primary anxiety disorder (absence of primary diagnosis) and recovery from all anxiety diagnosis (absence of any diagnoses). Predictors that were not significantly associated with either outcome at any time point were removed from further analyses. Significant associations were found for treatment group, age, symptom severity (from parent report), co-morbid behavior problems, GAD as primary disorder and presence of a Separation Anxiety Disorder (SAD) diagnosis. SPSS Generalized Estimating Equations (GEE) procedure was used to fit separate longitudinal regression models for each of these remaining predictor using unstructured correlation structure. GEE is robust to violations of normality and due to using population averaged parameters, is more flexible in the handling of missing data when within cluster numbers are small (Zorn, 2001), making it appropriate for our data. Each model included treatment intensity (full, brief) and time (post treatment, six month follow up), as well as the main effect of each predictor variable. Thus if a significant predictor of outcome was identified this was after adjustments for both the number of treatment sessions and time. In addition, in order to test whether predictors were specific to a particular time point, each model also included time-by-predictor interaction effects.

## 3 Results

### 3.1 Predictors of recovery from primary diagnosis

**Fig. 1:**
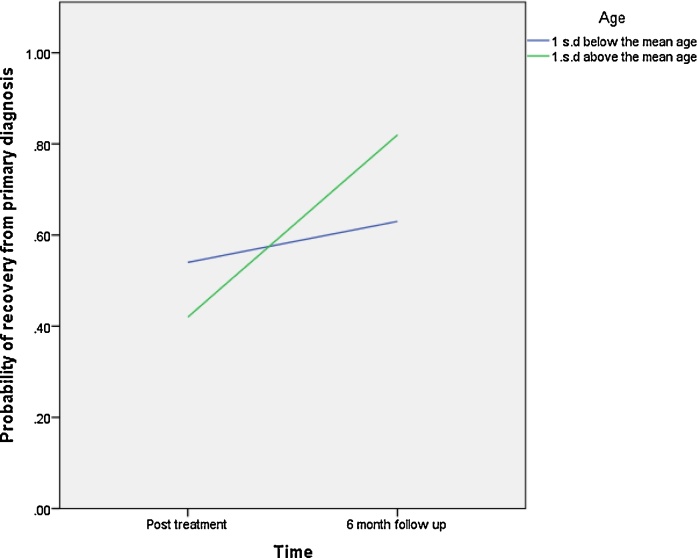
Child Age × Time interaction on probability of recovery from primary diagnosis.

**Fig. 2:**
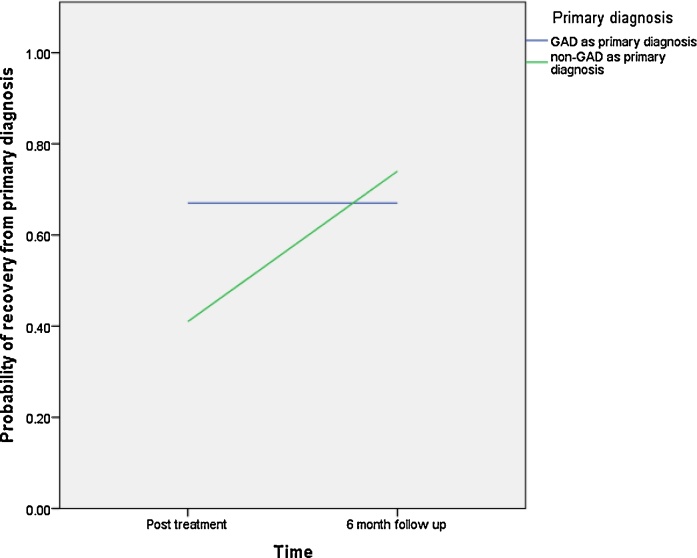
Primary diagnosis of GAD × Time interaction on probability of recovery form primary diagnosis.

As shown in Table 3, none of the predictor variables was significantly associated with recovery from primary diagnosis. However, significant time-by-predictor interaction effects were found for child age and primary diagnosis of GAD (time × age: β= 0.04, p = 0.00, OR = 1.04; time × GAD: (β= 1.57, p = 0.00, OR = 4.82)). Specifically younger children were more likely than older children to be free of their primary diagnosis post treatment, whereas older children showed a more favorable outcome at 6 months (see Fig. 1). Children who had GAD as their primary anxiety disorder had higher rates of recovery for their primary diagnosis at post treatment compared to those children with other primary anxiety disorders, but they had lower rates of recovery at six month follow-up. Interestingly, rates of recovery from primary diagnosis did not change from post treatment to six month follow up in children with GAD as their primary disorder, whereas children with other presentations made further gains within this time period (see Fig. 2).

### 3.2 Predictors of recovery from all anxiety diagnoses

**Fig. 3:**
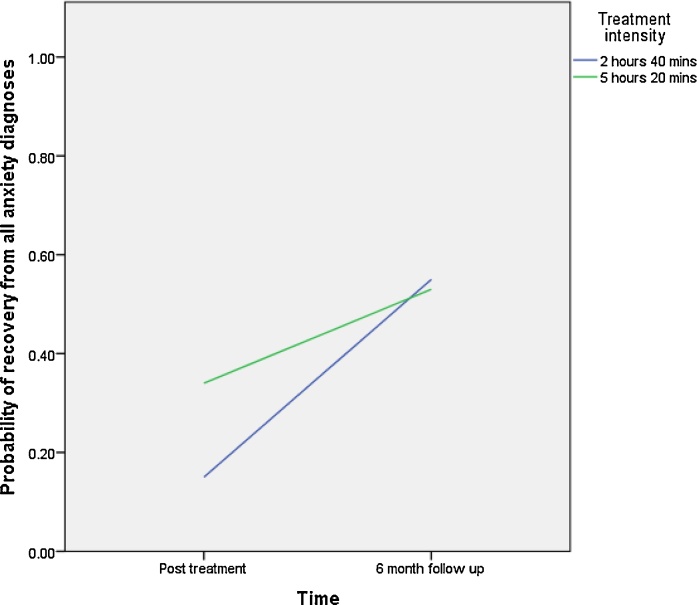
Treatment intensity × Time interaction for probability of recovery from all anxiety diagnoses.

As shown in Table 3, no main effects were found for any of the predictor variables on recovery from all anxiety diagnoses. However significant time-by-predictor interaction effects were found for treatment intensity (β = 0.98, p =0.04, OR = 2.66). As shown in Fig. 3, children whose parents received less guidance (2 h 40 min) had lower rates of recovery post treatment compared to those who had 5 h 20 min of guidance, but outcomes were very similar by 6 months.

## 4 Discussion

Consistent with the broader literature, where there is an absence of reliable predictors of treatment success in treatments for child anxiety disorders (Knight et al., 2014; Lundkvist-Houndoumadi et al., 2014), none of the 15 variables investigated here significantly predicted treatment outcomes. However, some interesting differences were found when the trajectory of treatment response was considered, and these have implications for decision making about ‘stepping-up’ children following low intensity interventions.

One of the clearest implications from the current findings is that if a child has a primary diagnosis of GAD and s/he has not recovered from that diagnosis immediately following treatment, then it is unlikely that s/he will recover and further treatment should be considered. On the other hand, children with the other primary diagnoses included here (most commonly separation anxiety disorder and social anxiety disorder) may make continued gains over the six months following treatment, so it may be worth monitoring their progress before offering further treatment. A similar recommendation could be made in relation to child age, where, if significant gains have not been made by the end of this low intensity treatment then further intervention should be considered for younger children, whereas older children may continue to make gains spontaneously.

It is also important to note that, although treatment intensity was associated with treatment outcome in the shorter-term, by the six month follow-up there were no significant differences in child treatment outcome on the basis of whether the parent was allocated to 2 h 40 min or 5 h and 20 min of therapist guidance. Health economic analyses will be important to establish whether the impact on the child and the family’s quality of life indicate the application of the somewhat more intensive treatment, in light of the costs associated with additional support (e.g. in academic, family and social domains) that might be incurred where there is a slower treatment response.

Further investigation is needed to determine whether the pattern of findings reported are found for child-focused CBT generally or are a particular feature of parent-delivered treatment. For example, it may be the case that parents find it easier to quickly implement strategies with their younger children, whereas it may take longer to engage their older children. It is also possible that parents find it harder to continue to make therapeutic gains for offspring with distress-based diagnoses (such as GAD) without therapist guidance than they would do for fear-based disorders (such as separation anxiety disorder and social anxiety disorder) due to the potentially less straightforward application of CBT techniques to overcome worry (Dugas & Koerner, 2005) and possible differences in the underlying mechanisms involved in fear versus distress based anxiety disorders in children (Waters, Bradley, & Mogg, 2014).

This study is the first to examine predictors of outcome in anxious children receiving GPD-CBT. The sample was derived from a larger randomized control trial (Thirlwall et al., 2013) and included children representative of a broad range of anxiety disorders and levels of impairment. The study further benefitted from high levels of assessment reliability and rigorous checks of treatment fidelity. The use of multiple outcome measures, across multiple time points allowed for examination of associations between predictors and outcome and the application of longitudinal regression analyses made it possible to investigate the interaction between predictors and the trajectory of recovery over time. A number of limitations, must also be considered, in particular the fact that the trial excluded children whose primary caregiver had a current anxiety disorder, a group known to have relatively unfavorable outcomes (Bodden, Bogels, & Nauta, 2008; Cobham, Dadds, & Spence, 1998), and, as such, findings cannot be generalized to the full population of children with anxiety disorders and replication of the study. Despite the advantages of using GEE analyses for our data, the model uses a population averaged method rather than a subject specific one. As such, the values of the regression coefficient are likely to be smaller than those found in other models. Our follow-up was limited to six months post-treatment, so, while we found that rates of recovery varied across different time points, the patterns of response beyond six months are not known. As we were interested in predictors of outcome at six months post treatment, a control group was not available so we cannot conclude that the associations were specific to treatment outcome rather than simply relating to naturalistic change over time. Finally, although this is the first evaluation of predictors of treatment outcomes for guided parent-led CBT for child anxiety disorders, studies which have examined predictors of child-focused CBT have typically failed to replicate each other’s findings. Further studies are clearly required to establish the generalizability of the current findings, as well as to explore additional predictors that may be particularly relevant to parent-led interventions, such as parental marital status and level of education.

## 5 Conclusion

In summary, child gender, age, symptom severity, co-morbidity and diagnostic category did not significantly predict outcomes following GPD-CBT. Older children and those offered only 2 h 40 min GPD-CBT may take longer to recover, but can be expected to make improvements by six months post treatment. Children with primary GAD initially respond favorably to GPD-CBT, but do not experience continuation of gains after treatment is complete. Future studies are needed to examine other possible predictors of outcome, such as parent characteristics, as well as the processes involved in change, in order to further enhance and tailor treatment for this population.

## Conflict of interest

CC receives royalties from the book used in this treatment.

CC receives royalties for the book provided to parents.

## Figures and Tables

**Table 1:** Descriptive Statistics for Predictors.

Continuous variables	N	Mean	SD
Age (months)	125	120	19.35
SCAS-C total	117	38	17.12
SCAS-P total	115	38	17.34
SMFQ total	117	7	5.15

Dichotomous variables	N	Frequency	Percentage
Gender (female)	125	60	48.0
Treatment group (brief))	125	61	48.8
Co-morbid anxiety	125	95	76.0
Co-morbid behavior problems	125	28	22.4
Primary diagnosis of SoPh	125	23	18.4
Primary diagnosis of SAD	125	30	24.0
Primary diagnosis of GAD	125	32	25.6
Any diagnosis of SoPh	125	75	60.0
Any diagnosis of SAD	125	60	48.0
Any diagnosis of GAD	125	72	57.6

*Note*: SoPh = primary diagnosis of social phobia; SAD = primary diagnosis of separation anxiety disorder; GAD = primary diagnosis of generalized anxiety disorder; SCAS-C = Spence Children’s Anxiety Scale, Child Version; SCAS-P = Spence Children’s Anxiety Scale, Parent version; SMFQ = Short Mood and Feelings Questionnaire.

**Table 2:** Correlations for Candidate Predictors and Treatment Outcomes at Both Time Points.

	Recovery form primary anxiety		Free of all anxiety	
	Post treatment	6 month follow-up	Post treatment	6 month follow-up
	*_χ_*^2^*_(p)_*	*_χ_*^2^*_(p)_*	*_χ_*^2^*_(p)_*	*_χ_*^2^*_(p)_*
Age in months	0.12 (0.27)	−0.21 (0.05)*	0.03 (0.78)	−0.05 (0.66)
SCAS-P total	0.26 (0.01)*	0.21 (0.05)*	0.29 (0.01)*	0.18 (0.10)
SCAS-C total	0.03 (0.81)	0.18 (0.11)	0.19 (0.07)	0.09 (0.42)
Mood	0.04 (0.65)	0.12 (0.24)	0.07 (0.50)	−0.01 (0.91)

	Recovery form primary anxiety		Free of all anxiety	
	Post treatment	6 month follow-up	Post treatment	6 month follow-up
	r_pb_*_(p)_*	r_pb_*_(p)_*	r_pb_*_(p)_*	r_pb_*_(p)_*
Gender	3.41 (0.07)	0.11 (0.74)	0.68 (0.41)	0.00 (0.95)
Treatment group	0.70 (0.40)	0.54 (0.46)	4.5 (0.03)*	0.04 (0.84)
Co-morbid anxiety	3.50 (0.06)	3.31 (0.07)		
Co-morbid behavior problems	4.04 (0.04)*	0.91 (0.34)	1.60 (0.21)	0.00 (0.99)
Primary diagnosis of SoPh	1.52 (0.22)	1.38 (0.24)	0.03 (0.87)	0.86 (0.36)
Primary diagnosis of SAD	0.28 (0.60)	0.26 (0.61)	0.02 (0.89)	2.04 (0.15)
Primary diagnosis of GAD	5.29 (0.02)*	0.46 (0.50)	0.43 (0.51)	0.70 (0.41)
Any diagnosis of SoPh	1.38 (0.24)	1.17 (0.28	0.91 (0.34)	0.04 (0.84)
Any diagnosis of SAD	1.66 (0.20)	1.17 (0.28)	4.57 (0.03)*	1.76 (0.18)
Any diagnosis of GAD	0.23 (0.63)	0.01 (0.91)	0.91 (0.34)	0.21 (0.64)

*Note*: * indicates statistically significant the 0.05 level; SCAS-C = Spence Children’s Anxiety Scale, Child Version; SCAS-P = Spence Children’s Anxiety Scale, Parent version; Treatment group = 4 sessions of GPD-CBT or 8 sessions of GPD-CBT; SoPh = primary diagnosis of social phobia; SAD = primary diagnosis of separation anxiety disorder; GAD = primary diagnosis of generalized anxiety disorder; df = 1.

**Table 3:** Predictors of Treatment Outcomes for GPD-CBT.

	Recovery from Primary diagnosis			Recovery from all anxiety diagnoses		
	B	p	OR	B	p	OR
Treatment group	0.35	0.45	1.42	−0.04	0.93	0.96
Treatment group * Time	−0.11	0.82	0.90	0.98	0.04*	2.66
Age	−0.02	0.06	0.97	−0.00	0.81	1.0
Age * Time	0.04	0.00**	1.04	0.01	0.41	1.01
SCAS-P total	0.03	0.06	1.03	0.02	0.09	1.02
SCAS-P total * Time	0.01	0.66	1.01	0.03	0.29	1.02
Co-morbid behavior problems	−0.75	0.21	0.47	−0.08	0.89	0.92
Co-morbid behavior problems * Time	−0.33	0.58	0.71	−0.75	0.33	0.48
Primary diagnosis of GAD	−0.43	0.40	0.65	0.40	0.43	1.49
Primary diagnosis of GAD * Time	1.57	0.00**	4.82	−0.02	0.97	0.98
Any diagnosis of SAD	0.50	0.29	1.65	0.63	0.14	1.88
Any diagnosis of SAD * Time	0.09	0.86	1.09	0.52	0.31	1.68

*Note*: *indicates statistically significant the 0.05 level; ** indicates statistically significant the 0.01 level: Treatment group = 4 sessions of GPD-CBT or 8 sessions of GPD-CBT; SCAS-P = Spence Children’s Anxiety Scale, Parent version: SoPh = primary diagnosis of social phobia; SAD = primary diagnosis of separation anxiety disorder; GAD = primary diagnosis of generalized anxiety disorder.

## References

[bib0005] Angold A., Costello E.J., Messer S.C. (1995). Development of a short questionnaire for use in epidemiological studies of depression in children and adolescents. International Journal of Methods in Psychiatric Research.

[bib0010] Berman S.L., Weems C.F., Silverman W.K., Kurtines W.M. (2000). Predictors of outcome in exposure-based cognitive and behavioral treatments for phobic and anxiety disorders in children. Behavior Therapy.

[bib0015] Bittner A., Egger H., Erkanli A. (2007). What do childhood anxiety disorders predict?. Journal of Child Psychology and Psychiatry.

[bib0020] Bodden D., Bogels S., Nauta M. (2008). Child versus family cognitive behavioural therapy in clinically anxious youth: An efficacy and partial effectiveness study. Journal of Clinical Child Psychology.

[bib0025] Bower P., Gilbody S. (2005). Stepped care in psychological therapies: Access, effectiveness and efficiency Narrative literature review. British Journal of Psychiatry.

[bib0030] Chavira D.A., Drahota A., Garland A.F. (2014). Feasibility of two modes of treatment delivery for child anxiety in primary care. Behaviour Research and Therapy.

[bib0035] Cobham V.E., Dadds M.R., Spence S.H. (1998). The role of parental anxiety in the treatment of childhood anxiety. Journal of Consulting and Clinical Psychology.

[bib0040] Cobham V.E., Dadds M.R., Spence S.H. (2010). Parental anxiety in the treatment of childhood anxiety: A different story three years later. Journal of Clinical Child Psychology.

[bib0045] Cobham V.E. (2012). Do anxiety-disordered children need to come into the clinic for efficacious treatment?. Journal of Consulting and Clinical Psychology.

[bib0050] Compton S.N., Peris T.S., Almirall D. (2014). Predictors and moderators of treatment response in childhood anxiety disorders: Results from the CAMS trial. Journal of Consulting and Clinical Psychology.

[bib0055] Creswell C., Willetts L. (2007). Overcoming your child's fears and worries: A self-help guide using cognitive-behavioural techniques.

[bib0060] Dugas M.J., Koerner N. (2005). Cognitive-behavioral treatment for generalized anxiety disorder: Current status and future directions. Journal of Cognitive Psychotherapy.

[bib0065] Ezpeleta L., Keeler G., Erkanli A. (2001). Epidemiology of psychiatric disability in childhood and adolescence. Journal of Child Psychology and Psychiatry.

[bib0070] Farmer E.M.Z., Stangl D.K., Burns B.J. (1999). Use, persistence, and intensity: Patterns of care for children's mental health across one year. Community Mental Health Journal.

[bib0075] Ford T., Goodman R., Meltzer H. (2003). The british child and adolescent mental health survey 1999: the prevalence of DSM-IV. Journal of the American Academy of Child and Adolescent Psychiatry Disorders.

[bib0080] Hudson J.L., Rapee R.M., Deveney C. (2009). Cognitive-behavioral treatment versus an active control for children and adolescents with anxiety disorders: A randomized trial. Journal of the American Academy of Child and Adolescent Psychiatry.

[bib0085] Hudson J.L., Keers R., Roberts S. (2015). Clinical predictors of response to cognitive-behavioral therapy in pediatric anxiety disorders: The genes for treatment (GxT) study. Journal of the American Academy of Child and Adolescent Psychiatry.

[bib0090] James A., James G., Cowdrey F.A. (2013). Cognitive behavioural therapy for anxiety disorders in children and adolescents. The Cochrane Library.

[bib0095] Kendall P.C., Hudson J.L., Gosch E. (2008). Cognitive-behavioral therapy for anxiety disordered youth: A randomized clinical trial evaluating child and family modalities. Journal of Consulting and Clinical Psychology.

[bib0100] Kessler R.C., Chiu W.T., Demler O. (2005). Prevalence, severity, and comorbidity of 12-month DSM-IV disorders in the national comorbidity survey replication (Vol. 62, p. 617, 2005). Archives of General Psychiatry.

[bib0105] Knight A., McLellan L., Jones M., Hudson J.L. (2014). Pre-treatment predictors of outcome in childhood anxiety disorders: A systematic review. Psychopathology Review.

[bib0110] Last C.G., Hansen C., Franco N. (1998). Cognitive‐behavioral treatment of school phobia. Journal of the American Academy of Child and Adolescent Psychiatry.

[bib0115] Leong J., Cobham V.E., De Groot J. (2009). Comparing different modes of delivery. European Child and Adolescent Psychiatry.

[bib0120] Liber J.M., van Widenfelt B.M., van der Leeden A.J. (2010). The relation of severity and comorbidity to treatment outcome with cognitive behavioral therapy for childhood anxiety disorders. Journal of Abnormal Child Psychology.

[bib0125] Lundkvist-Houndoumadi I., Hougaard E., Thastum M. (2014). Pre-treatment child and family characteristics as predictors of outcome in cognitive behavioural therapy for youth anxiety disorders. Nordic Journal of Psychiatry.

[bib0130] Lyneham H.J., Rapee R.M. (2006). Evaluation of therapist-supported parent-implemented CBT for anxiety disorders in rural children. Behaviour Research and Therapy.

[bib0135] Nauta M.H., Scholing A., Rapee R.M. (2004). A parent-report measure of children’s anxiety: Psychometric properties and comparison with child-report in a clinic and normal sample. Behaviour Research and Therapy.

[bib0140] Polanczyk G.V., Salum G.A., Sugaya L.S. (2015). Annual research review: A meta-analysis of the worldwide prevalence of mental disorders in children and adolescents. Journal of Child Psychology and Psychiatry.

[bib0145] Rapee R., Abbott M., Lyneham J. (2006). Bibliotherapy for children with anxiety disorders using written materials for parents: A randomized controlled trial. Journal of Consulting and Clinical Psychology.

[bib0150] Rapee R.M., Lyneham H.J., Hudson J.L., Kangas M., Wuthrich V.M., Schniering C.A. (2013). Effect of comorbidity on treatment of anxious children and adolescents: Results from a large, combined sample. Journal of the American Academy of Child and Adolescent Psychiatry.

[bib0155] Reynolds S., Wilson C., Austin J. (2012). Effects of psychotherapy for anxiety in children and adolescents: A meta-analytic review. Clinical Psychology Review.

[bib0160] Salloum A. (2010). Minimal therapist-assisted cognitive–behavioral therapy interventions in stepped care for childhood anxiety. Professional Psychology: Research and Practice.

[bib0165] Sharp C., Goodyer I.M., Croudace T.J. (2006). The Short Mood and Feelings Questionnaire (SMFQ): A unidimensional item response theory and categorical data factor analysis of self-report ratings from a community sample of 7-through 11-year-old children. Journal of Abnormal Child Psychology.

[bib0170] Silverman W.K., Albano A.M. (1996). The anxiety disorders interview schedule for DSM-IV – child and parent versions.

[bib0175] Silverman W.K., Saavedra L.M., Pina A.A. (2001). Test-retest reliability of anxiety symptoms and diagnoses with the anxiety disorders interview schedule for DSM-IV: Child and parent versions. Journal of the American Academy of Child and Adolescent Psychiatry.

[bib0180] Smith A.M., Flannery-Schroeder E.C., Gorman K.S. (2014). Parent cognitive-behavioral intervention for the treatment of childhood anxiety disorders: A pilot study. Behaviour Research and Therapy.

[bib0185] Spence S.H. (1998). A measure of anxiety symptoms among children. Behaviour Research and Therapy.

[bib0190] Stallard P., Udwin O., Goddard M. (2007). The availability of cognitive behaviour therapy within specialist child and adolescent mental health services (CAMHS): A national survey. Behavioural and Cognitive Psychotherapy.

[bib0195] Thirlwall K., Cooper P.J., Karalus J. (2013). Treatment of child anxiety disorders via guided parent-delivered cognitive-behavioural therapy: Randomised controlled trial. British Journal of Psychiatry.

[bib0200] Walkup J.T., Albano A.M., Piacentini J. (2008). Cognitive behavioral therapy, sertraline, or a combination in childhood anxiety. The New England Journal of Medicine.

[bib0205] Waters A.M., Bradley B., Mogg K. (2014). Biased attention to threat in paediatric anxiety disorders (generalized anxiety disorder, social phobia, specific phobia, separation anxiety disorder) as a function of ‘distress’ versus ‘fear’diagnostic categorization. Psychological Medicine.

[bib0210] Zorn C.J. (2001). Generalized estimating equation models for correlated data: A review with applications. American Journal of Political Science.

